# ﻿Confirmation of the valid specific status of *Dolichovespulakuami* Kim & Yoon, 1996 (Hymenoptera, Vespidae) based on molecular and morphological evidence

**DOI:** 10.3897/zookeys.1196.110224

**Published:** 2024-03-22

**Authors:** Chang-Jun Kim, Jiang-Li Tan, Jeong Kyu Kim, Moon Bo Choi

**Affiliations:** 1 Division of Gardens and Education, Korea National Arboretum, Pocheon, 11186, Republic of Korea Korea National Arboretum Pocheon Republic of Korea; 2 Key Laboratory for Animal Conservation / Key Laboratory of Resource Biology and Biotechnology in Western China, College of Life Sciences, Northwest University, Xi’an, Shaanxi 710069, China Northwest University Xi'an China; 3 Department of Bio Environment Health, Dongnam Health University, Suwon, 16328, Republic of Korea Dongnam Health University Suwon Republic of Korea; 4 Institute of Plant Medicine, Kyungpook National University, Daegu, 41566, Republic of Korea Kyungpook National University Daegu Republic of Korea; 5 Department of R&D, Wild Beei, Chilgok, 39864, Republic of Korea Department of R&D, Wild Beei Chilgok Republic of Korea

**Keywords:** Description, DNA barcoding, *Dolichovespulaflora*, male, mt-COI, taxonomy, Vespidae, wasp

## Abstract

The taxonomic validity of *Dolichovespulakuami*, especially in relation to *D.flora*, has been the subject of a long-term debate. Herein, the valid specific status of the former was supported through an integrated analysis of morphological characters and DNA barcodes. The pronotal rugae and male genitalia of the two species are different, and partial mitochondrial genes (cytochrome oxidase subunit I, COI) indicate that they form significantly distinct lineages. The hitherto unknown male of *D.kuami* is described for the first time, and a brief discussion of the *D.maculata* species group is provided.

## ﻿Introduction

To date, 19 species in the genus *Dolichovespula* Rohwer, 1916 (Hymenoptera: Vespidae) have been described from the Palaearctic, Nearctic and Oriental regions (Archer 2012; Tan et al. 2014; Daglio 2020; Wang et al. 2022). Among these, the *Dolichovespulamaculata* group includes a total of four species (*D.flora* Archer, 1987, *D.maculata* (Linnaeus, 1763), *D.media* (Retzius, 1783) and *D.kuami* Kim & Yoon, 1996), the members of which are characterized by the presence of pronotal striae (or furrows), the structure of the male aedeagus, and a strongly notched seventh gastral sternum (Archer 2012). Within this group, the taxonomic validity of *D.kuami* with respect to *D.flora* remains a matter of contention (Archer 1999, 2006, 2012). Whereas Tan et al. (2014) considered these to be conspecific taxa, Kim (2011) has presented evidence to indicate that *D.kuami* is a valid discrete species. This discrepancy can be attributed, at least in part, to a lack of information regarding male characteristics and differences in interpreting the related morphological variations. In 2014, however, Tan et al. described the males of *D.flora* after discovering their nests. Recently, two of the authors of this study (JKK and MBC) identified two young and mature nests of *D.kuami* in Korea and obtained specimens of all relevant castes in 2018 and 2019, respectively.

In an attempt to resolve the longstanding debate surrounding the taxonomic validity of *D.kuami* and *D.flora*, in this study, we performed further morphological comparisons and DNA barcode analyses (using the partial mt-COI gene). We also thoroughly re-examined the orientation of the pronotal rugae and the structure of the male genitalia, and compared DNA barcodes of the two species to assess their genetic limits. A description of the previously unrecorded male of *D.kuami* is also provided.

## ﻿Materials and methods

### ﻿Morphological terminology

The terminology used in this study follows that described by Archer (1987, 1999).

### ﻿Illustrations

The images were captured using a Leica DFC 495 camera mounted on a Leica M205A stereozoom microscope (Leica Microsystems, Solms, Germany) and acquired by using LAS v.4.1.0 (Leica Microsystems, Switzerland). In addition, to observe the pronotal rugae of *D.kuami* and *D.flora*, scanning electron microscopy (SEM) images of specimens selected from each species were obtained. Subsequently, the head, metasoma, wings and legs were removed and only the mesosoma was retained. The separated mesosomes were immersed in 10% NaOCl to remove excess tissue. The detached mesosoma was washed with distilled water using a soft brush to remove the remaining tissues. After cleaning, the samples were dried and coated with gold ion particles for SEM observation (Hitachi SU8220 & SU8230, Tokyo, Japan). Image plates were prepared using Adobe Photoshop CS6 (Adobe Systems Inc., San Jose, CA, USA).

### ﻿DNA extraction and amplification

Genomic DNA was extracted from the hind legs of dried or 100% alcohol-preserved specimens using the DNeasy Blood and Tissue Kit (Qiagen) after incubating for 24 h at 56 °C in lysis buffer and Proteinase K, as per the manufacturer’s instructions. A total of 26 specimens of *D.kuami*, eight specimens of *D.media*, and two specimens of *D.flora* were sequenced. The cytochrome c oxidase I barcoding region was amplified using the primer pair LepF1 and LepR1 (Hebert et al. 2004). PCR was performed using Solg^TM^ 2X Taq PCR Pre-Mix (Solgent). We prepared 30 μL of reaction mixture containing 15 μL of PCR Pre-Mix, 11 μL of nuclease-free dH_2_O, 2 μL DNA template, and 1 μL of 10 pmol of each primer. A typical PCR program started with 4 min of initial denaturation at 94 °C, followed by 40 cycles of 30 s at 94 °C, 45 s of annealing at 45 °C, and 45 s of elongation at 72 °C, ending with a 6-min period of final elongation at 72 °C.

### ﻿Molecular phylogenetic analyses

Sequence reads were edited and assembled using Geneious 11 (Biomatters, Auckland, New Zealand). The optimal model (GTR+I+G) for each partition was selected using the Akaike information criterion in jModelTest (Posada 2008). The tree was constructed using the maximum-likelihood method with RAxML v.8.1.2 (Stamatakis 2014), with 1000 bootstrap replications. Successful sequences were submitted to the NCBI GenBank (accession numbers presented in Table 1).

**Table 1.** Specimens, vouchers and GenBank accession information of *Dolichovespula* species included in the molecular phylogenetic analysis.

**Species**	**Specimens**	**Vouchers**	**GenBank Accession** #
1. *Dolichovespulakuami* Kim & Yoon, 1996	Korea: Gyeonggi-do, Pocheon-si	20Ves0603	OR029465
	Korea: Gyeonggi-do, Pocheon-si	20Ves0604	OR029466
	Korea: Gyeonggi-do, Yangpyeong-si	20Ves0605	OR029467
	Korea: Gangwon-do, Hwacheon-gun	20Ves0606	OR029468
	Korea: Gangwon-do, Hwacheon-gun	20Ves0607	OR029469
	Korea: Gyeonggi-do, Cheorwon-gun	20Ves0608	OR029470
	Korea: Gyeonggi-do, Pocheon-si	20Ves0609	OR029471
	Korea: Gangwon-do, Hwacheon-gun	20Ves0610	OR029472
	Korea: Gyeonggi-do, Namyangju-si	20Ves0611	OR029473
	Korea: Gyeonggi-do, Yeoju-gun	20Ves0613	OR029474
	Korea: Gyeonggi-do, Namyangju-si	20Ves0614	OR029475
	Korea: Gyeonggi-do, Yangju-si	20Ves0615	OR029476
	Korea: Gangwon-do, Hwacheon-gun	20Ves0616	OR029477
	Korea: Gyeonggi-do, Yangpyeong-si	20Ves0617	OR029478
	Korea: Gyeonggi-do, Yangpyeong-si	20Ves0619	OR029479
	Korea: Gyeonggi-do, Pocheon-si	20Ves0621	OR029480
	Korea: Gyeonggi-do, Pocheon-si	20Ves0622	OR029481
	Korea: Gyeonggi-do, Paju-si	20Ves0623	OR029482
	Korea: Gyeonggi-do, Yangpyeong-si	20Ves0624	OR029483
	Korea: Gangwon-do, Yanggu-gun	20Ves0627	OR029484
	Korea: Gyeonggi-do, Yeoju-gun	20Ves0628	OR029485
	Korea: Gangwon-do, Yanggu-gun	20Ves0630	OR029486
	Korea: Gyeonggi-do, Pocheon-si	20Ves0631	OR029487
	Korea: Gyeonggi-do, Pocheon-si	20Ves0632	OR029488
	Korea: Gyeonggi-do, Pocheon-si	20Ves0633	OR029489
	Korea: Gyeonggi-do, Pocheon-si	20Ves0635	OR029490
2. *D.media* (Retzius, 1783)	Korea: Gangwon-do, Yanggu-gun	20Ves0594	OR029457
	Korea: Gangwon-do, Goseong-gun	20Ves0595	OR029458
	Korea: Gangwon-do, Yanggu-gun	20Ves0596	OR029459
	Korea: Gangwon-do, Goseong-gun	20Ves0597	OR029460
	Korea: Gangwon-do, Hwacheon-gun	20Ves0598	OR029461
	Korea: Gangwon-do, Yanggu-gun	20Ves0599	OR029462
	Korea: Gangwon-do, Yanggu-gun	20Ves0600	OR029463
	Korea: Gangwon-do, Goseong-gun	20Ves0601	OR029464
3. *D.flora* Archer, 1987	China: Shaanxi, Huangbaiyuan	20Ves0643	OR029491
	China: Shaanxi, Huaxian	China004	OR029492
4. *D.maculata* (Linnaeus, 1763)	GenBank search	GenBank	KU874876
	GenBank search	GenBank	KJ147231
5. *D.norwegica* (Fabricius, 1781)	GenBank search	GenBank	KU874880
6. *D.alpicola* (Wagner, 1978)	GenBank search	GenBank	KM568773
7. *D.norvegicoides* (Sladen, 1918)	GenBank search	GenBank	MG374965
8. *D.saxonica* (Fabricius, 1793)	GenBank search	GenBank	KJ147234
9. *D.pacifica* (Birula, 1930)	GenBank search	GenBank	KJ147233
10. *D.arenarina* (Fabricius, 1775)	GenBank search	GenBank	KJ147230
11. *D.adulterina* (Buysson, 1905)	GenBank search	GenBank	KM567260
12. *D.sylvestris* (Scopoli, 1763)	GenBank search	GenBank	KJ147235
13. *Vespacrabro* Linnaeus, 1758	GenBank search	GenBank	KJ147244
14. *Formicafusca* Linnaeus, 1758	GenBank search	GenBank	LT977378

In addition, for phylogenetic analysis, other *Dolichovespula* species and outgroups, excluding the *D.maculata* group (*D.flora*, *D.maculata*, *D.media* and *D.kuami*), included were as follows: KU874880 (*Dolichovespulanorwegica*), KM568773 (*D.alpicola*), MG374965 (*D.norvegicoides*), KJ147234 (*D.saxonica*), KJ147233 (*D.pacifica*), KJ147230 (*D.arenarina*), KM567260 (*D.adulterina*), KJ147235 (*D.sylvestris*), KJ147244 (*Vespacrabro*) and LT977378 (*Formicafusca*) (Table 1). The latter two species, from Vespidae and Formicidae, respectively, were included to test the monophyly of the family and root the tree, respectively.

### ﻿Specimens

Thirty-five specimens were used in this study to review the taxonomic positions of the focus species, *D.kuami* (25) from the Korea National Arboretum (Pocheon, Republic of Korea) and *D.flora* (2) from Northwest University (Xi’an, China), and *D.media* (8).

## ﻿Results

### ﻿Comparison of the pronotal rugae and carina of *D.kuami* and *D.flora*

**Figure 1.**
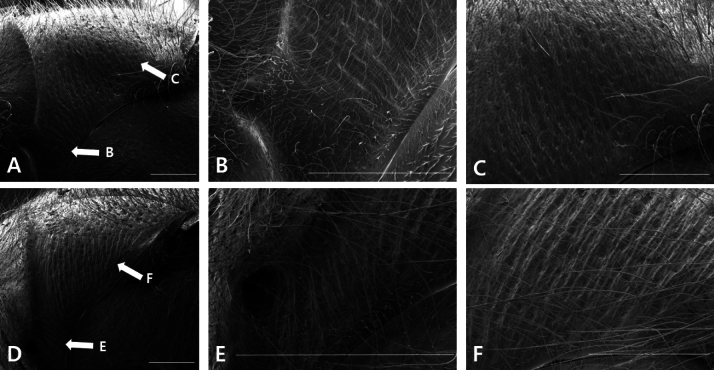
Comparison between the pronotal rugae and carinas of *D.kuami* and *D.flora*. Pronotum of *D.kuami* (**A**) and *D.flora* (**D**); rugae and carinas in the pronotal lateral part to the pronotal pit of *D.kuami* (**B**) and *D.flora* (**E**); rugae on the pronotum of *D.kuami* (**C**) and *D.flora* (**F**). Arrows in figures **A, D** indicate the enlarged parts in **B, C, E, F**. Scale bars: 0.5 mm.

The pronotum rugae of *D.kuami* were generally very dull and faint (Fig. 1A), whereas those of *D.flora* were relatively more distinct (Fig. 1D). We found that *D.kuami* has faint longitudinal rugae on the pronotal lateral face next to the pronotal pit (Fig. 1B), and the remaining posterior area has fine rugae running vertically (Fig. 1C). On the other hand, *D.flora* has distinct longitudinal rugae (Fig. 1E) that run downward, except in the upper pronotal area (Fig. 1F). Thus, there is a clear difference between the pronotal rugae of these two species. Additionally, the pronotum carina was sharper in *D.kuami* than in *D.flora*.

### ﻿Comparison of the external genitalic features of male *D.kuami* and *D.flora*

**Figure 2.**
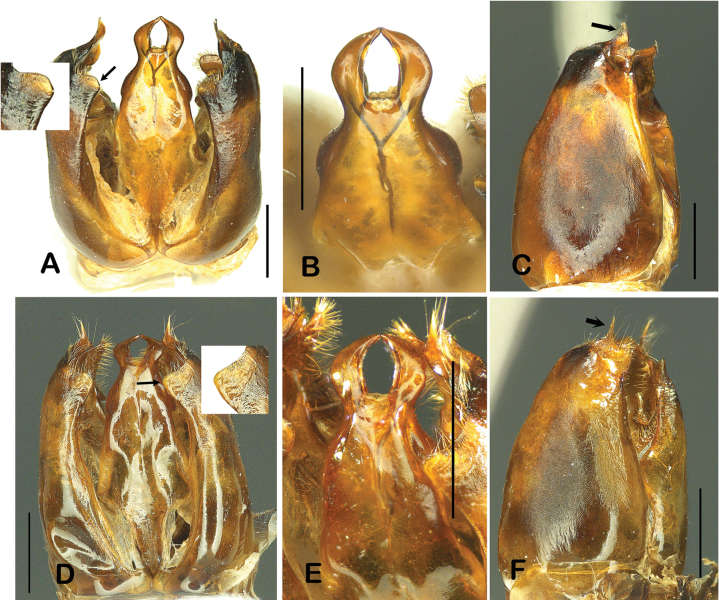
Genitalia of *Dolichovespulakuami* (**A–C**) and *D.flora* (**D–F**) **A** genitalic capsules, in dorsal view (truncated dorsal terminal process, arrow) **B** aedeagus **C** gonostipes and triangular parameral spine (arrow) **D** genitalic capsules, in dorsal view (shortly rounded terminal process, arrow) **E** aedeagal tip **F** gonostipes and slender parameral spine (arrow). Scale bars: 1 mm.

Genitalia (Fig. 2A–F). The external features of the genitalia of *D.kuami* and *D.flora* are very similar (Fig. 2A, D; also refer to Tan et al. 2014 for *D.flora*). However, *D.kuami* had a triangular parameral spine (Fig. 2C, arrow), whereas that of *D.flora* is short and slender (Fig. 2F, arrow). In addition, *D.kuami* has a truncated dorsal terminal process (Fig. 2A, arrow), whereas it is somewhat edged or shortly rounded in *D.flora* (Fig. 2D, arrow).

### ﻿DNA barcoding

**Figure 3.**
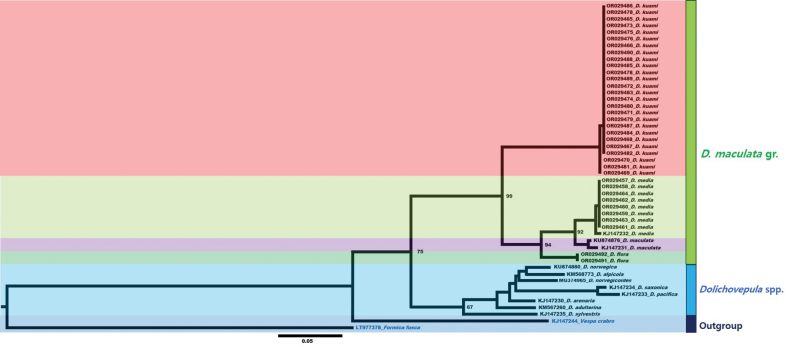
A maximum-likelihood phylogenetic tree of the successfully DNA barcoded *Dolichovespula* specimens. The numbers above the branches indicate bootstrap proportions.

Phylogenetically, *D.maculata* clearly clustered with other *Dolichovespula* spp. (Fig. 3). In the *D.maculata* group, *D.flora* was more closely related to *D.maculata* and *D.media*, whereas *D.kuami* clustered as a sister species to the clade that included these three species (Fig. 3). Thus, the two species *D.flora* and *D.kuami* stat. rev. were clearly separated into well-supported clusters, and the genetic distance between them was relatively high (average DNA barcode distance: 0.0996), suggesting that they represent two biological species. These results were further supported by those of the morphological examination.

## ﻿Discussion

According to Archer (1999), *D.kuami* was treated as a conspecific to *D.flora* because the orientation of the rugae in the lateral part of the pronotum is the same, and the observed differences in body color are a type of variation. Although Kim (2011) re-described this aspect with additional specimens, Archer (2012) consistently insisted that these two species were conspecific. In fact, as shown by Kim (2011) (Fig. 2), the rugae of the pronotum were not clearly distinguished under light microscopy because they are the same color as the base color. Therefore, in this study, we attempted to obtain very clear rugae images using SEM and observed that the pronotal rugae of the two species were clearly different. Despite these morphological differences, differences in male genitalia or DNA sequences are most critically needed to provide evidence of the difference between these two species (Archer 1999; Tan et al. 2014).

**Figure 4.**
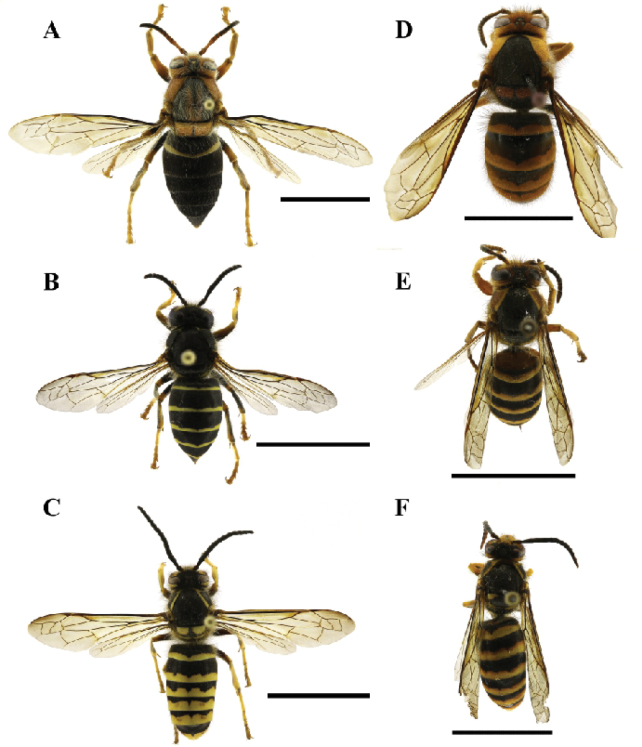
Comparison of the general habitus of *Dolichovespulakuami* and *D.flora* (**A, D** queen **B, E** worker **C, F** drone): *D.kuami* (**A–C**) *D.flora* (**D–F**). Scale bars: 1 cm.

*Dolichovespulakuami* and *D.flora* are uncommon species in Korea and China, respectively, and their nests and males have not been recorded for many years. Tan et al. (2014) collected males of *D.flora* and described their genitalia. We collected males for the first time in 2018, when the first nest of *D.kuami* was discovered. This discovery enabled us to compare the male genitalia of the two species. In general, their external morphologies were relatively similar, but there were clear differences in the parameral spine and dorsal terminal processes. None of the *D.kuami* strains were conspecific to *D.flora* based on evidence of their pronotal rugae, color patterns (Fig. 4), genitalia and DNA sequences. These results support the conclusion of the long-term conspecific debate and the specific status of *D.kuami*.

Additionally, DNA barcoding is an excellent tool for accelerating species identification and complementing species delimitation (Mo et al. 2021; Zhang and Wenjun 2022; Jafari et al. 2023). In particular, COI barcode information from the genus *Polistes*, a related genus of Vespidae, provides insight into the phylogenetic relationships within the group (Schmid-Egger et al. 2017). Based on the results of this study, morphological evidence and DNA barcoding in Vespidae will provide critical evidence to resolve species delimitations in the future.

## ﻿Additional information

### Author ORCIDs

Chang-Jun Kim https://orcid.org/0000-0002-5823-8703

Jeong Kyu Kim https://orcid.org/0000-0003-2175-5798

### Data availability

All of the data that support the findings of this study are available in the main text.

## References

[B1] Archer ME (1987) Three new species of *Dolichovespula* (Hym., Vespidae) from China. Entomologist’s Monthly Magazine 123: 27–31.

[B2] Archer ME (1999) Taxonomy and world distribution of the Euro-Asian species of *Dolichovespula* (Hym., Vespidae). Entomologist’s Monthly Magazine 135: 153–160.

[B3] Archer ME (2006) Taxonomy, distribution and nesting biology of species of the genus *Dolichovespula* (Hymenoptera, Vespidae). Entomological Science 9(3): 281–293. 10.1111/j.1479-8298.2006.00174.x

[B4] Archer ME (2012) Vespine wasp of the world behavior, ecology, taxonomy of the Vespinae. Siri Scientific Press. Manchester, UK.

[B5] Daglio A (2020) Yellowjackets of the World. LAP LAMBERT Academic Publishing. Mauritius.

[B6] Hebert PDN Penton EH Burns JM Janzen DH Hallwachs W (2004) Ten species in one: DNA barcoding reveals cryptic species in the neotropical skipper butterfly *Astraptesfulgerator*. Proceedings of the National Academy of Sciences of the United States of America 101(41): 14812–14817. 10.1073/pnas.0406166101 15465915 PMC522015

[B7] Jafari S Müller B Rulik B Rduch V Peters RS (2023) Another crack in the Dark Taxa wall: A custom DNA barcoding protocol for the species-rich and common Eurytomidae (Hymenoptera, Chalcidoidea). Biodiversity Data Journal 11: e101998. 10.3897/BDJ.11.e101998 PMC10189536 37206111

[B8] Kim JK (2011) *Dolichovespulakuami* (Vespidae, Hymenoptera): Taxonomic complement with newly found specimens. Sociobiology 57: 11–18.

[B9] Kim JK Yoon IB (1996) A new species of *Dolichovespula* (Insecta: Hymenoptera: Vespidae) from Korea. Korean Journal of Systematic Zoology 12: 199–202.

[B10] Mo W-h Chen H-y Pang H Liu J-x (2021) DNA barcoding for molecular identification of the genus *Oxyscelio* (Hymenoptera, Scelionidae) from southern China, with descriptions of five new species. In: Lahey Z Talamas E (Eds) Advances in the Systematics of Platygastroidea III.Journal of Hymenoptera Research 87: 613–633. 10.3897/jhr.87.7191

[B11] Posada D (2008) jModelTest: phylogenetic model averaging. Molecular Biology & Evolution 25(7): 1253–1256. 10.1093/molbev/msn083 18397919

[B12] Schmid-Egger C van Achterberg K Neumeyer R Morinière J Schmidt S (2017) Revision of the West Palaearctic Polistes Latreille, with the descriptions of two species – an integrative approach using morphology and DNA barcodes (Hymenoptera, Vespidae). ZooKeys 713: 53–112. 10.3897/zookeys.713.11335 PMC5674218 29134040

[B13] Stamatakis A (2014) RAxML version 8: A tool for phylogenetic analysis and post-analysis of large phylogenies. Bioinformatics 30(9): 1312–1313. 10.1093/bioinformatics/btu033 24451623 PMC3998144

[B14] Tan JL Chen X van Achterberg C (2014) Description of the male *Dolichovespulaflora* Archer (Hymenoptera: Vespidae). Entomotaxonomia 36(1): 75–80. 10.3897/zookeys.391.6606

[B15] Wang H Wen Q Wang T Ran F Wang M Fan X Wei S Li Z Tan J (2022) Next-Generation Sequencing of Four Mitochondrial Genomes of *Dolichovespula* (Hymenoptera: Vespidae) with a Phylogenetic Analysis and Divergence Time Estimation of Vespidae. Animals (Basel) 12(21): 3004. 10.3390/ani12213004 36359128 PMC9657509

[B16] Zhang H Wenjun B (2022) Exploring Large-Scale Patterns of Genetic Variation in the COI Gene among Insecta: Implications for DNA Barcoding and Threshold-Based Species Delimitation Studies. Insects 13(5): 425. 10.3390/insects13050425 35621761 PMC9147995

